# Supplementary material for: Directional matching of swimming polarity provides a competitive advantage during bacterial magneto-aerotaxis
Source: BMC Microbiol. 2026 May 1;26:449. doi: 10.1186/s12866-026-05067-8 (PMC13154873; doi:10.1186/s12866-026-05067-8)
Supplement: Supplementary file 1 — Supplementary Material 1. [file 12866_2026_5067_MOESM1_ESM.pdf]

Additional materials for

Directional Matching of Swimming Polarity Provides a Competitive Advantage During Bacterial Magneto-Aerotaxis

Carina Weigel and Daniel Pfeiffer

Corresponding author: Daniel Pfeiffer
Email: daniel.pfeiffer@uni-bayreuth.de

This PDF file includes:

Figures S1 to S10

Other additonal materials for this manuscript include the following:

Tables S1 to S2
Legendes for Movies S1 to S4
Movies S1 to S4

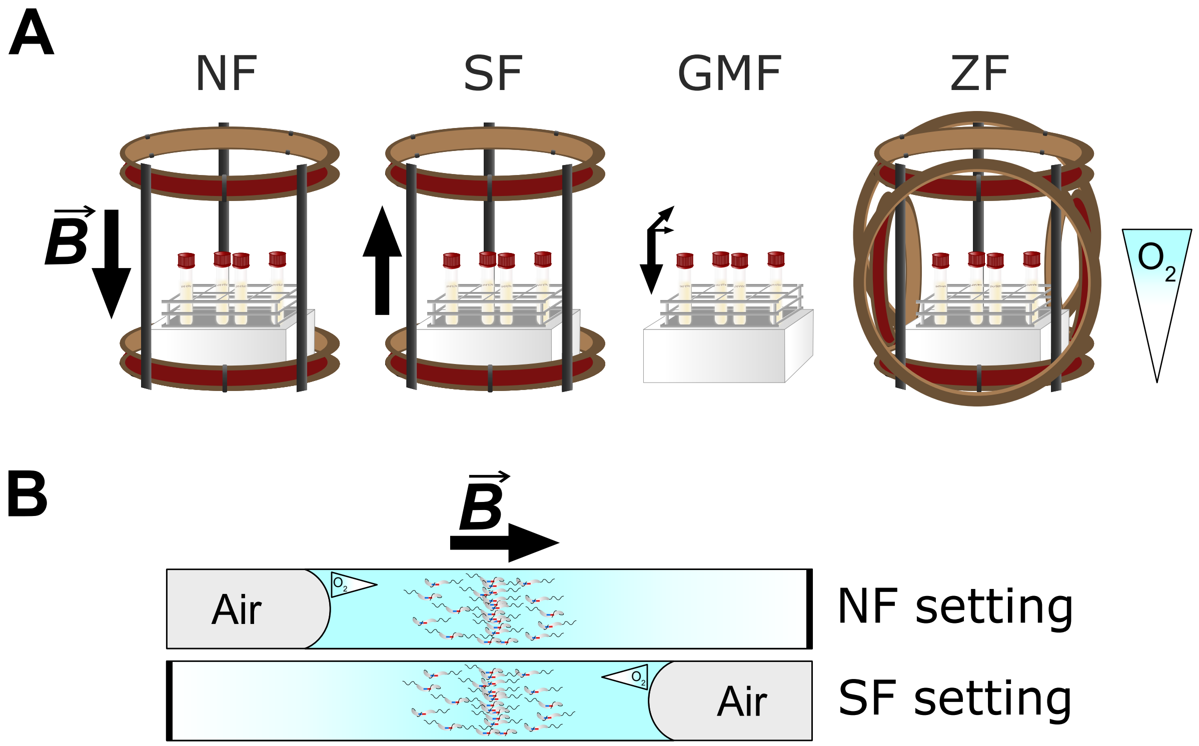

Fig. S1. Magnetic field configurations used during culture incubation and in capillary assays. (A) Cultures were grown under four different magnetic field conditions in non-agitated culture tubes, allowing an oxygen gradient to form: NF: Northern Field, a uniform 0.6 mT field mimicking Northern Hemisphere polarity (generated by coils). SF: Southern Field, a uniform 0.6 mT field mimicking Southern Hemisphere polarity (generated by coils). GMF: The local geomagnetic field in Bayreuth, Germany (north, east, and vertical components of 20.0 μT, 1.5 μT, and 45.0 μT, respectively; horizontal field ≈ 20.1 μT; total magnitude ≈ 49.3 μT). ZF: A zero field produced by triaxial coil pairs. **(B)** To compare cell behavior under magnetic fields resembling Northern- and Southern-Hemisphere geomagnetic field polarity (referred to as NF and SF setting, respectively), two capillaries filled with cell suspensions were placed side-by-side, with one rotated by 180° to align the aerotactic bands along the microscope stage x-axis under a homogeneous 400 μT magnetic field. Black arrows indicate the field direction and polarity, and gradient-filled triangles show the direction of oxygen gradients.

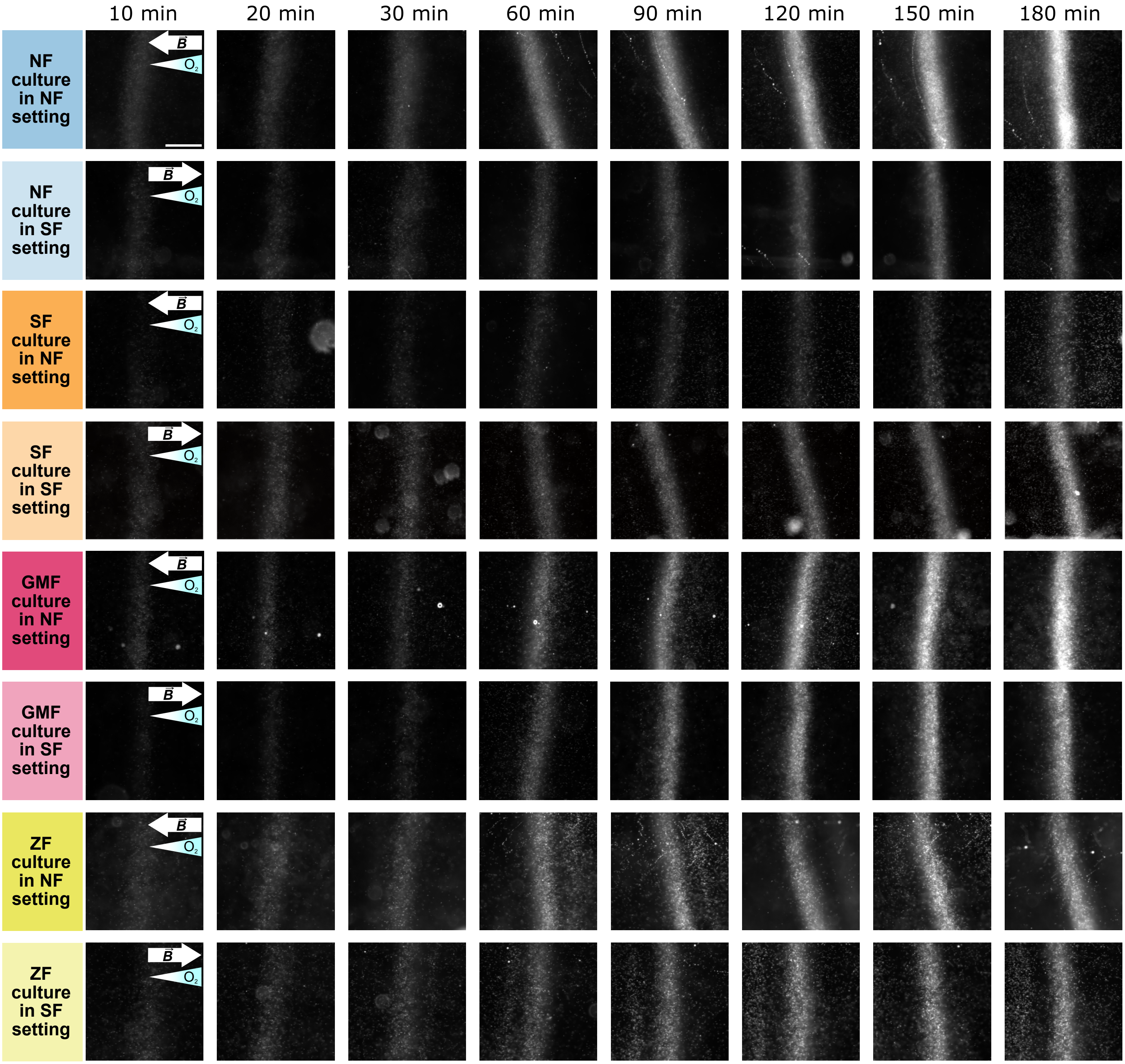

Fig. S2. Temporal progression during aerotactic band formation. Shown are representative dark-field micrographs of wild-type aerotactic bands at different time points under NF and SF settings for samples obtained from different magnetic-field preculturing conditions. In particular, the later time points show pronounced differences in band intensity between matching and non-matching magnetic field settings. Cell counting yielded a range of approximately 4.3–6.3 × 10^6 cells/cm^3 in the early aerotactic band at t = 10 min, based on the eight analyzed bands shown in this figure. Although this represents a rough estimate that is derived from different experimental conditions, it falls within a similar order of magnitude as MTB cell densities reported near oxic–anoxic transition zones in microcosms (Flies et al. 2005, FEMS Microbiology Ecology, 52:185–95). White arrows indicate magnetic field (*B*) directions; gradient-filled triangles indicate oxygen gradient directions. Scale bar: 100 µm (applies to all images; shown only in the upper leftmost image). Data correspond to **Figs. 2–4**.

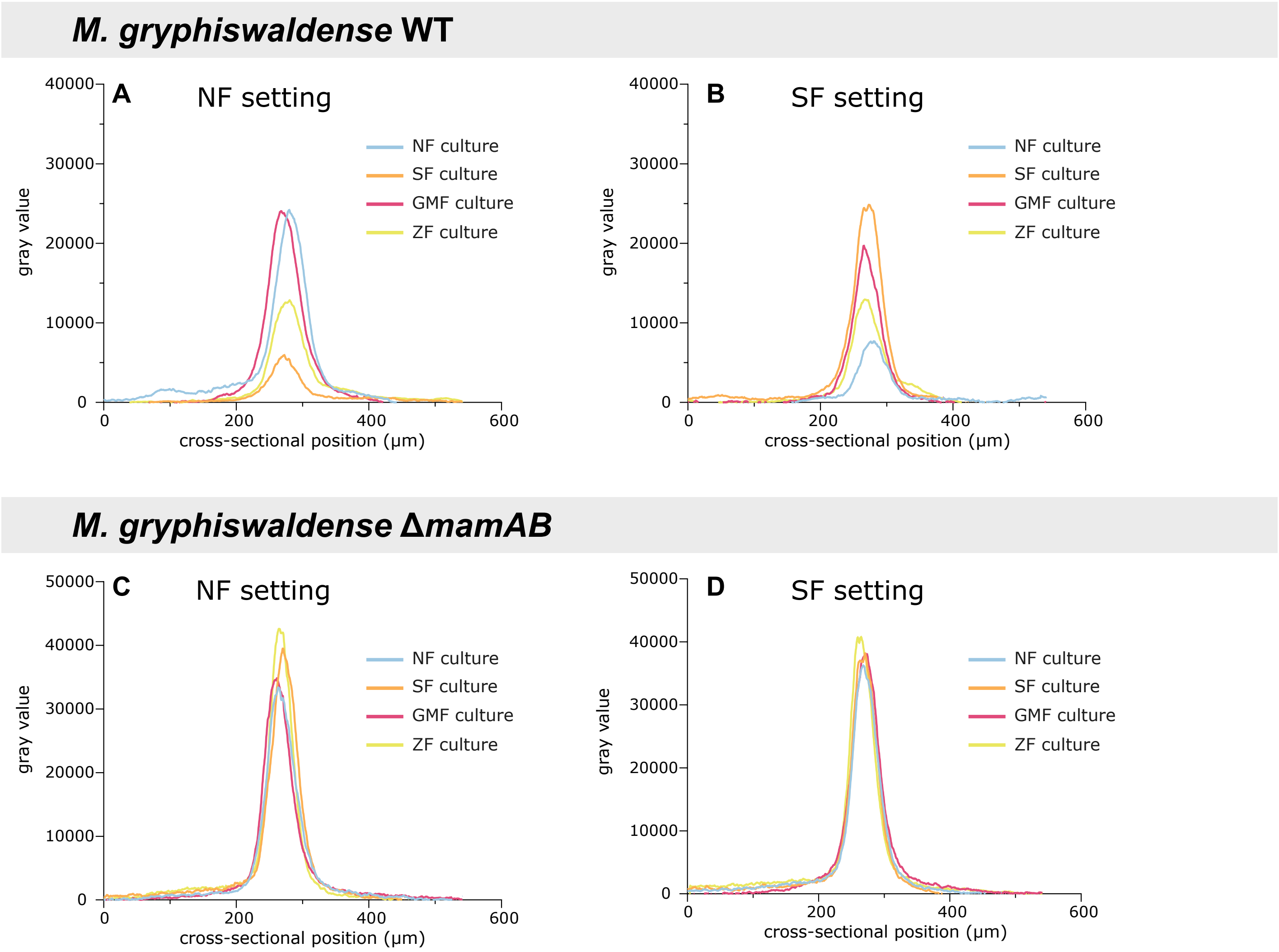

Fig. S3. Aerotactic band intensity profiles. Overlaid, averaged aerotactic band intensity profiles (after 180 min) from NF, SF, GMF, and ZF cultures of both the wild type **(A, B)** and the Δ*mamAB* strain **(C, D)** under NF **(A, C)** and SF **(B, D)** settings. Data correspond to **Figs. 2** and **4**.

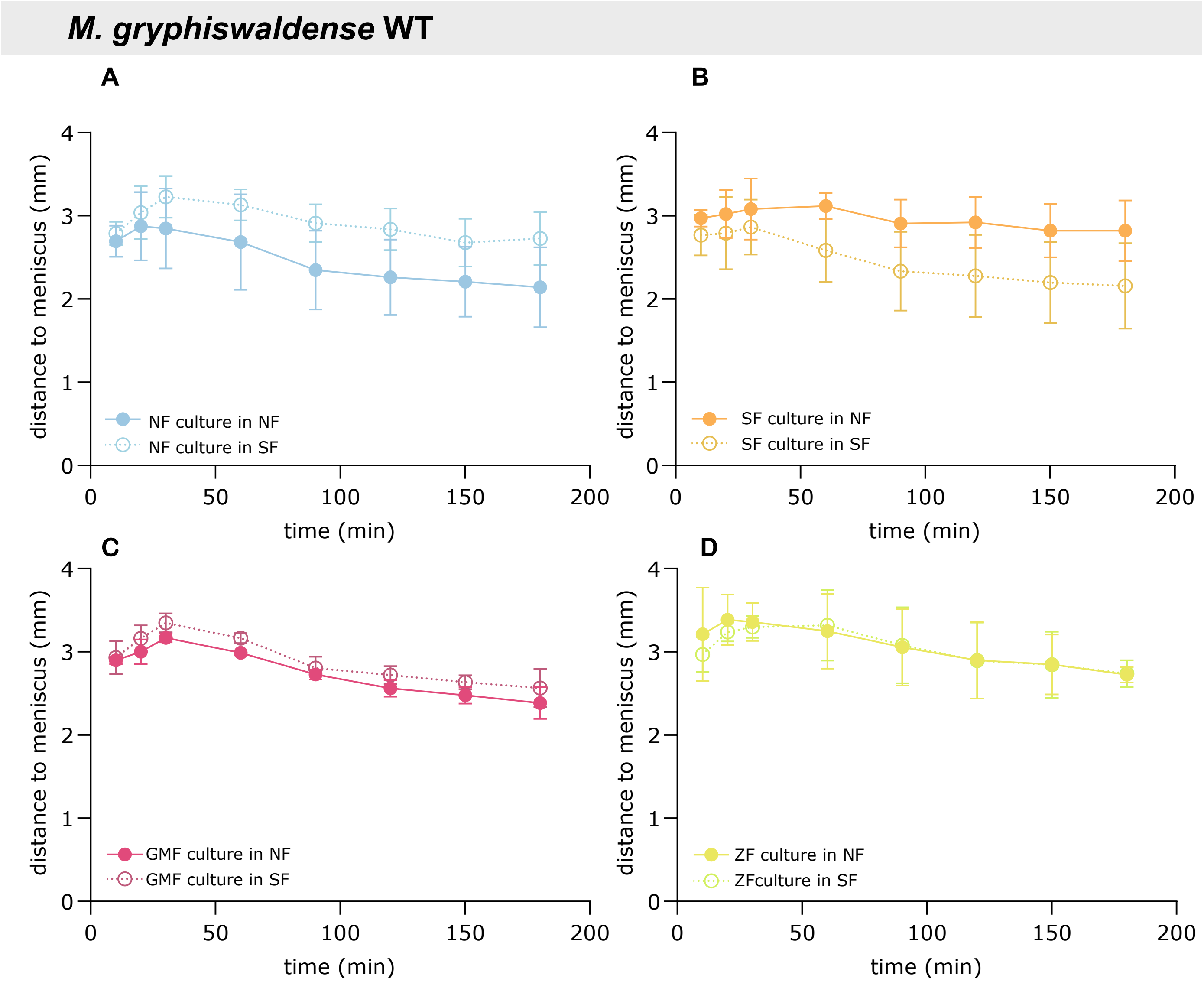

Fig. S4. Spatiotemporal dynamics of aerotactic band positioning for wild-type cultures. The mean distance (± SD) between the aerotactic band and the air-liquid interface (meniscus) was measured at defined time points (*n* = 3 capillary experiments). **(A)** NF culture; **(B)** SF culture; **(C)** GMF culture; **(D)** ZF culture—each analyzed under NF and SF settings (solid and dashed lines, respectively). Data correspond to **Figs. 2** and **3**.

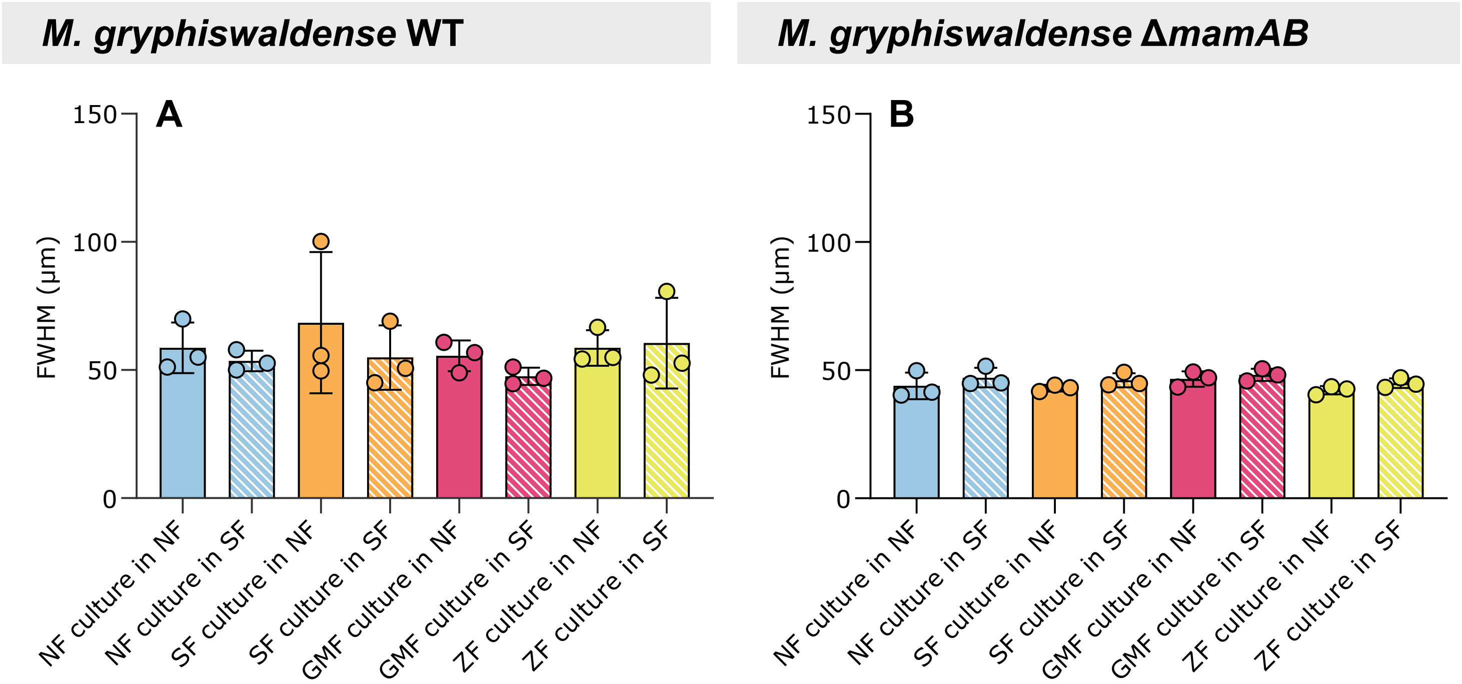

Fig. S5. Widths of aerotactic bands. The full width at half maximum (FWHM) of aerotactic bands was measured after 180 min for the wild-type **(A)** and Δ*mamAB* **(B)** strains under both NF and SF settings (colored and dashed bars, respectively). Bars represent the mean, error bars the SD, and individual measurements are shown as dots (n = 3 independent experiments). No statistically significant differences were found between samples or magnetic field conditions (Kruskal-Wallis test with Dunn's multiple-comparison test, $p \geq 0.05$). Data correspond to **Figs. 2–4**.

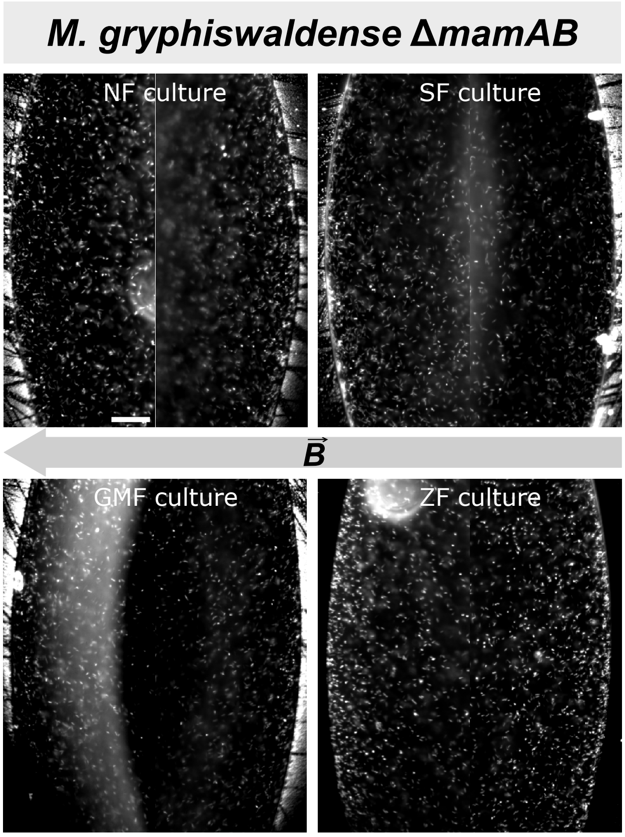

Fig. S6. Hanging-drop assay of Δ*mamAB* cultures. Cells were precultivated under the following magnetic field conditions: NF: uniform magnetic field applied parallel to the oxygen gradient; SF: uniform magnetic field applied antiparallel to the oxygen gradient; GMF: ambient geomagnetic field (Bayreuth, Germany); ZF: ambient magnetic fields canceled. Because Δ*mamAB* cells lack biomagnetism, swimming polarity is not selected during preculture, and in the hanging-drop assay cells do not preferentially accumulate at either drop edge facing magnetic north or south. The gray arrow indicates the magnetic field direction during the assay. The scale bar (50 µm) is shown in the top-left image and applies to all panels.

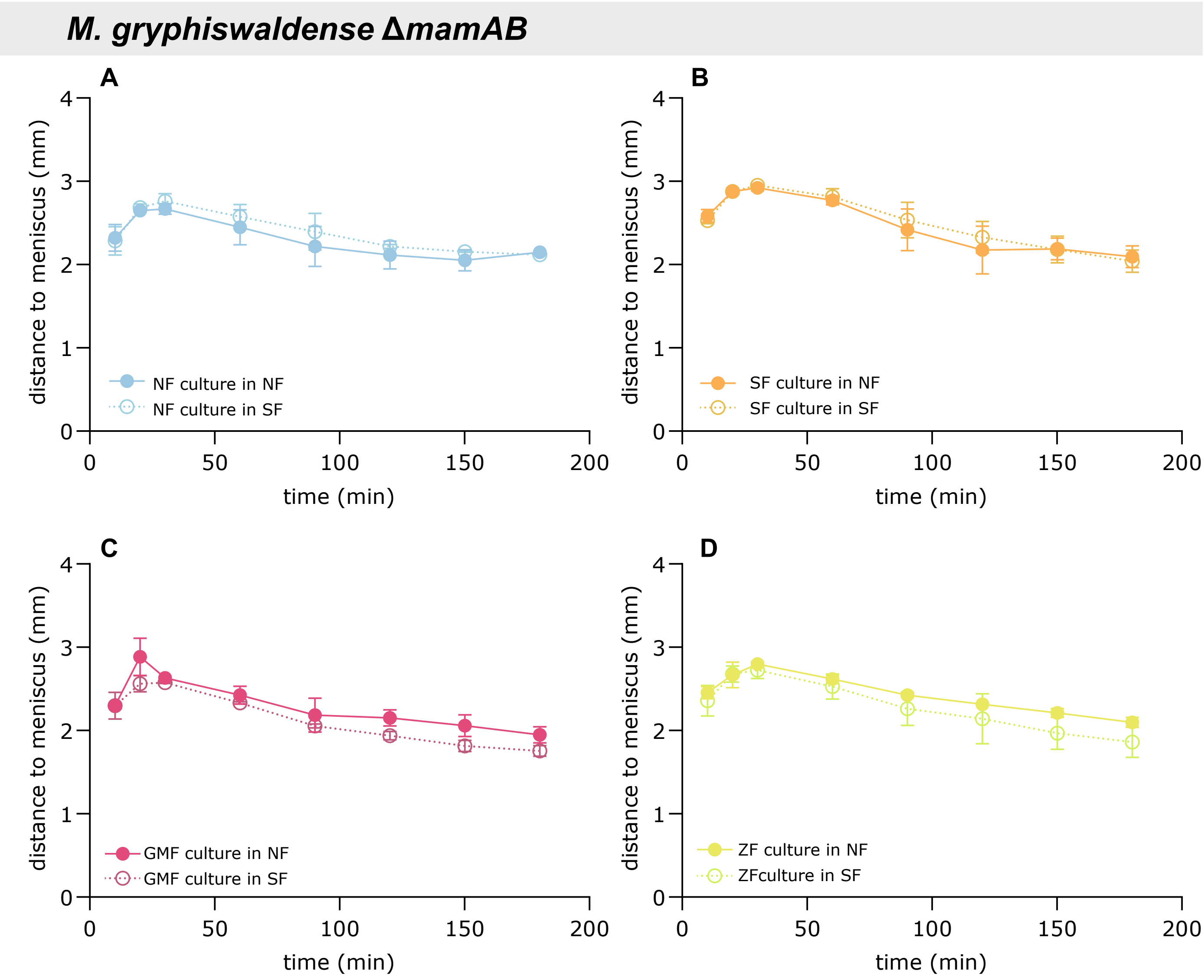

Fig. S7. Spatiotemporal dynamics of aerotactic band positioning for Δ*mamAB* cultures. The distance between the aerotactic band and the air-liquid interface (meniscus) was measured at defined time points. **(A)** NF culture; **(B)** SF culture; **(C)** GMF culture; **(D)** ZF culture—each analyzed under NF and SF settings (solid and dashed lines, respectively). Data represent the mean ± SD from *n* = 3 microcapillary experiments and correspond to **Fig. 4**.

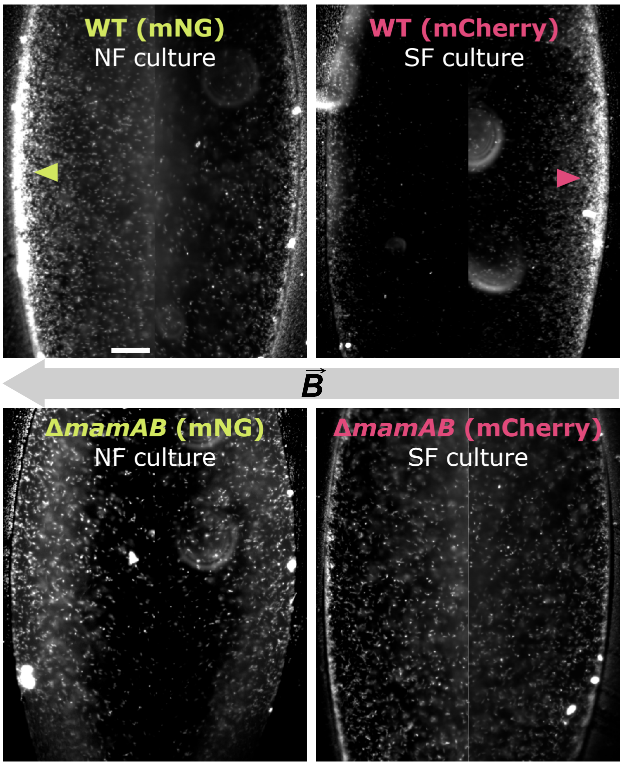

Fig. S8. Hanging-drop assay of fluorescently labeled wild-type and Δ*mamAB* cells from NF and SF cultures. mNG- and mCherry-labeled cells, precultivated under NF and SF conditions, respectively, were analyzed for swimming polarity in the hanging-drop assay before being mixed in equal amounts for competition assays. Dark-field micrographs are shown. The gray arrow indicates the magnetic field direction. Colored arrowheads indicate preferential enrichment of cells with the respective fluorescence label. The scale bar (50 μm) is depicted in the top-left image and applies to all panels. Data correspond to **Fig. 5**.

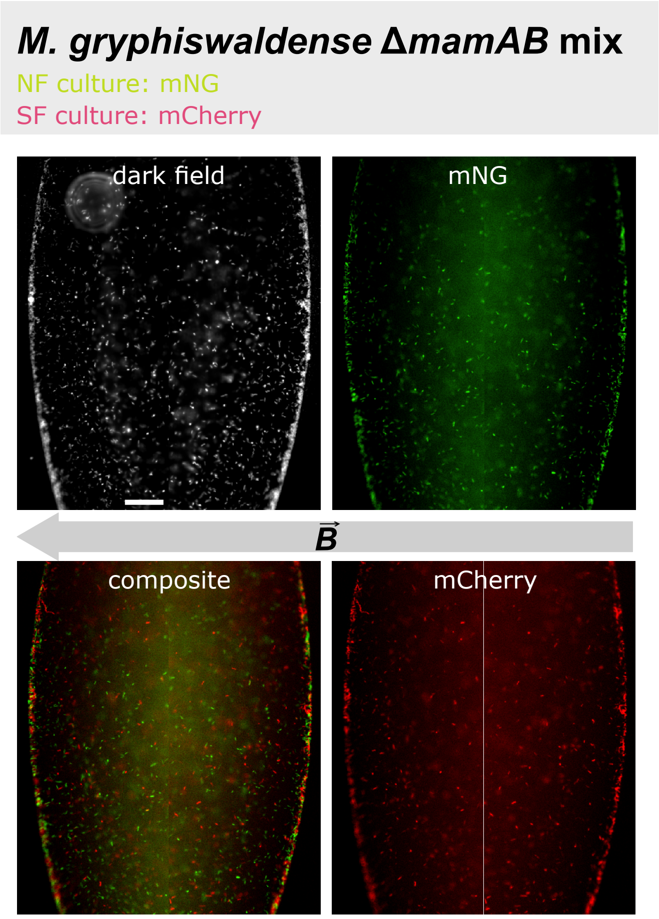

Fig. S9. Hanging-drop assay of fluorescently labeled Δ*mamAB* cells. mNG- and mCherry-labeled Δ*mamAB* cells, precultivated under NF and SF conditions, respectively, were analyzed in the hanging-drop assay after being mixed in equal amounts for competition assays. Dark-field image (upper row, left), mNG channel (upper row, right), composite of the mNG and mCherry channels (bottom row, left), and mCherry channel (bottom row, right) are shown. The gray arrow indicates the magnetic field direction. The scale bar (50 µm) is depicted in the top-left image and applies to all panels.

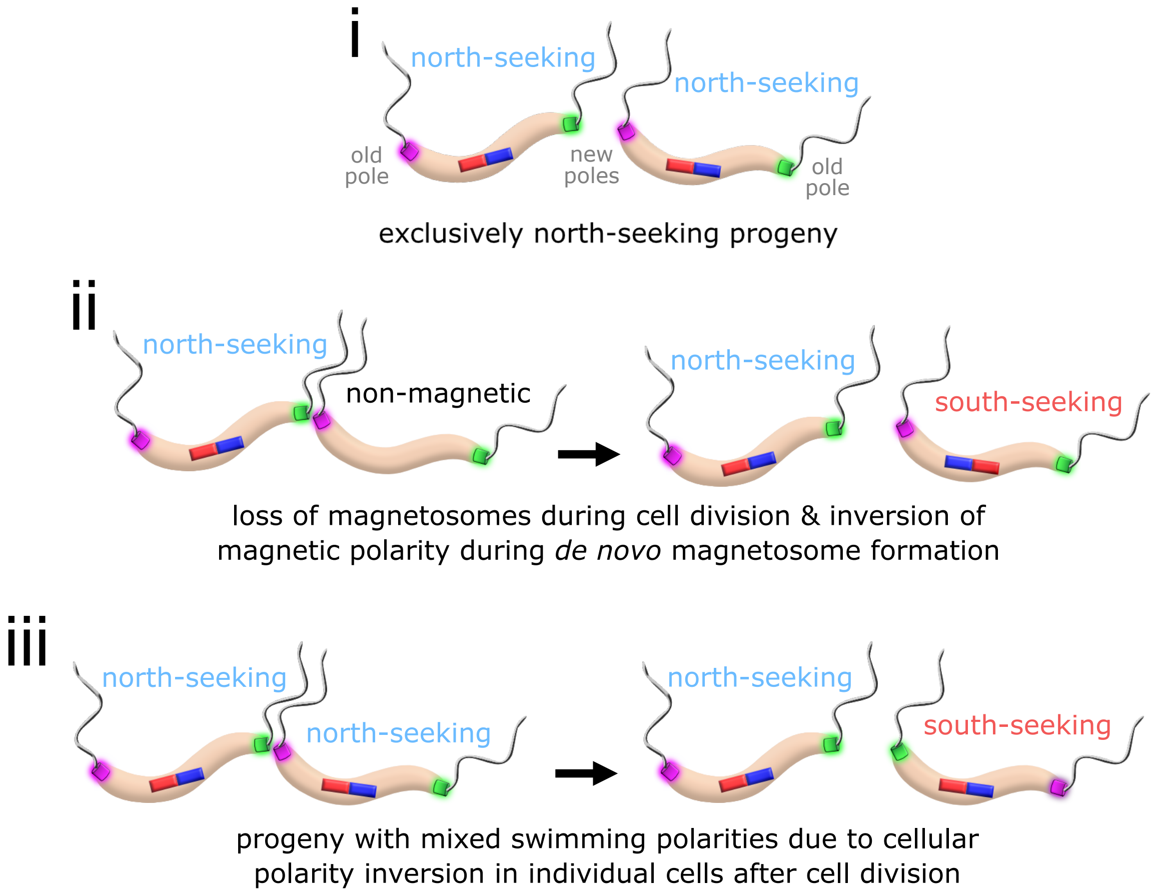

Fig. S10. Hypothetical mechanisms of swimming polarity inheritance and inversion in bipolarly flagellated magnetospirilla. (i) Swimming polarity may be maintained through the inheritance of a fixed positional arrangement of two flagellar motor subtypes relative to the net magnetic moment of the magnetosome chain. Note that, relative to the old and new cell poles (indicated in gray font), daughter cells exhibit opposing cellular polarities to account for their opposing magnetic polarities when magnetosome chains are split during cell division. **(ii)** If swimming polarity is maintained through cell division, swimming polarity inversion may occur due to random loss of magnetosomes during division (due to missegregation) and subsequent *de novo* magnetosome formation (i.e., inversion of magnetic polarity). **(iii)** Alternatively, swimming polarity inversion may occur in individual cells through an inversion of cellular polarity by an unknown mechanism. In this scenario, as illustrated in the example, the cell on the right would invert the identities of its motors—i.e., the purple motor would become a green motor, and the green motor would become a purple motor. Note that north- and south-seeking swimming polarity here refers to the overarching swimming-polarity type characteristic of the cells, and not only to the immediate observed swimming direction of cells, as in some studies, which depends on whether cells encounter oxygen levels above or below their optimum. This figure includes assumptions that have not yet been experimentally verified and is intended solely to illustrate potential mechanisms of swimming polarity inversion.
