# Supplementary material for: Directional matching of swimming polarity provides a competitive advantage during bacterial magneto-aerotaxis
Source: BMC Microbiol. 2026 May 1;26:449. doi: 10.1186/s12866-026-05067-8 (PMC13154873; doi:10.1186/s12866-026-05067-8)
Supplement: Supplementary file 2 — Supplementary Material 2. [file 12866_2026_5067_MOESM2_ESM.pdf]

Supplementary Tables

Table S1. Strains and plasmids. *E. coli* strains with plasmids are not individually listed.

Strain or vector	Relevant characteristic(s)	Reference and/or source
**Strains**		
E. coli		
DH5α	Host for cloning; F^- φ80*lacZ*ΔM15 Δ(*lacZYA-argF*)U169 *recA1 endA1 hsdR17*(r_K^- m_K^+) *phoA supE44* λ^- *thi-1 gyrA96 relA1*	[1]
WM3064	Conjugation strain; *thrB1004 pro thi rpsL hsdS lacZ*ΔM15 *RP4-1360* Δ(*araBAD*)*567* Δ*dapA1341*::[*erm pir*]	William Metcalf at UIUC
M. gryphiswaldense		
MSR-1 R3/S1	Laboratory wild type (WT); Rif^R, Sm^R	[2]
Δ*mamAB*	*mamAB* operon deletion strain	[3]
MSR-1 R3/S1 Tn*7*:: $P_{mamDC45}$-*omNG100*	mNeonGreen-producing wild-type strain; Km^R	This study
MSR-1 R3/S1 Tn*7*:: $P_{mamDC45}$-*mCherry*	mCherry-producing wild-type strain; Km^R	This study
Δ*mamAB* Tn*7*:: $P_{mamDC45}$-*omNG100*	mNeonGreen-producing Δ*mamAB* strain; Km^R	This study
Δ*mamAB* Tn7:: $P_{mamDC45}$-*mCherry*	mCherry-producing Δ*mamAB* strain; Km^R	This study
**Plasmids**		
pBAMII-Tn7	Site-specific (*glmS* locus) Tn*7*-based insertion vector; *tnsABCD*, Km^R, Amp^R	René Uebe at UBT (un-published)
pBAMII-$P_{mamDC45}$-*omNG100*	Vector for genomic insertion of $P_{mamDC45}$-*omNG100*	This study
pBAMII-$P_{mamDC45}$-*mCherry*	Vector for genomic insertion of $P_{mamDC45}$-*mCherry*	This study

Table S2. Oligonucleotides used for generating fluorescently labeled strains for competition experiments. Restriction sites used for cloning are underlined.

No.	Primer name	Sequence (5‘-3‘)
558	pBAMII-Tn7_fwd	GGAACTGCCTGGGCGAATTTAGC
559	pBAMII-Tn7_rev	AAATAGATGGGAACTGGGTGTAGCGTCG
717	PmamDC_SmaI/XmaI_fw2	ACATCCCCGGGGCGAATTCCTCGAGCTTTTTCGCTT
718	omNG100_NotI_rev	CCGGCGGCCGCTCACTTATACAGTTCGTCCATGCCC
719	mCherry_NotI_rev	CCGGCGGCCGCTTACTTGTACAGCTCGTCCATGCC

References

1. Hanahan D. Studies on Transformation of *Escherichia coli* with Plasmids. J Mol Biol. 1983;166:557–80. https://doi.org/10.1016/s0022-2836(83)80284-8.

2. Schultheiss D, Kube M, Schüler D. Inactivation of the Flagellin Gene *flaA* in *Magnetospirillum gryphiswaldense* Results in Nonmagnetotactic Mutants Lacking Flagellar Filaments. Appl Environ Microbiol. 2004;70:3624–31. https://doi.org/10.1128/AEM.70.6.3624-3631.2004.

3. Ullrich S, Schüler D. Cre-*lox*-Based Method for Generation of Large Deletions within the Genomic Magnetosome Island of *Magnetospirillum gryphiswaldense*. Applied and Environmental Microbiology. 2010;76:2439–44. https://doi.org/10.1128/AEM.02805-09.
